# Therapeutic effects of human umbilical cord mesenchymal stem cell-derived microvesicles on premature ovarian insufficiency in mice

**DOI:** 10.1186/s13287-019-1327-5

**Published:** 2019-08-14

**Authors:** Ziling Yang, Xin Du, Cunli Wang, Jin Zhang, Conghui Liu, Yu Li, Hong Jiang

**Affiliations:** 1 Reproductive Medicine Center, 105th Hospital of PLA, Hefei, 230031 Anhui People’s Republic of China; 2 The First affiliated Hospital of Anhui Medical University, Hefei, 230032 Anhui People’s Republic of China

**Keywords:** Mesenchymal stem cells (MSCs), Microvesicles (MVs), Premature ovarian insufficiency (POI), Angiogenesis, PI3K/AKT

## Abstract

**Background:**

Premature ovarian insufficiency (POI) is one of the leading causes of female infertility, which is caused by an abnormal ovarian reserve. Currently, there is no effective treatment to restore the fertility of POI patients. Recent studies suggested that microvesicles (MVs) released from mesenchymal stem cells (MSCs) exert therapeutic effects in various degenerative diseases. In this study, the effect of human umbilical cord MSC-derived MVs (HUCMSC-MVs) on the restoration of ovarian function in a chemotherapy-induced POI mouse model is investigated.

**Methods:**

MVs were obtained from the supernatant of cultured HUCMSCs. The localization of PKH26-labeled HUCMSC-MVs in ovarian tissues was observed by confocal laser scanning microscopy. Histomorphometric analysis was performed to count the number of ovarian follicles and vessels. The ovarian sections were stained with anti-CD34 to evaluate angiogenesis. The levels of estradiol (E2) and follicle-stimulating hormone (FSH) were measured by enzyme-linked immunosorbent serologic assay. The mRNA expression of angiogenesis-related cytokines and the protein expression of AKT in mouse ovaries were measured by quantitative RT-PCR and western blot analysis. The parametric variables were compared by Student’s *t* test and analysis of variance. The non-parametric variables were compared by the Mann-Whitney *U* test. Categorical variables were compared by *χ*^2^ test. *P* < 0.05 was considered statistically significant.

**Results:**

PKH26-labeled HUCMSC-MVs were detectable within the ovaries and migrated to the ovarian follicles 24 h after transplantation. The transplantation of HUCMSC-MVs could increase the body weight and number of ovarian follicles (primordial, developing, and preovulatory follicles), induce ovarian angiogenesis, and recover the disturbed estrous cycle of POI mice. The expression levels of total AKT, p-AKT, and angiogenic cytokines (including VEGF, IGF, and angiogenin) in the ovaries of POI mice were markedly upregulated after HUCMSC-MVs transplantation, suggesting that HUCMSC-MVs transplantation might recover ovarian function by inducing angiogenesis via the PI3K/AKT signaling pathway.

**Conclusions:**

This study provides valuable insight into the effects of HUCMSC-MVs on ovarian tissue angiogenesis and on the restoration of ovarian function in POI mice, which may be helpful to develop a treatment strategy for POI patients.

## Background

Premature ovarian insufficiency (POI) is a common cause of infertility and is characterized by hypoestrogenism and increased levels of gonadotropins, resulting from the exhaustion of the ovarian follicles. It affects 1–2% of women younger than 40 years of age and 0.1% of women younger than 30. Women with POI usually suffer from primary or secondary amenorrhea, infertility, hot flushes, sexual dysfunction, and/or vaginal dryness, leading to poor quality of life. Moreover, POI is a heterogeneous disorder of multi-factorial origin that is clinically irreversible, and currently, no proven effective therapy exists. Therefore, new and effective treatment strategies are urgently required [1–4].

Stem cells, which possess the characteristics of rapid self-renewal, indefinite proliferative capacity, multi-directional differentiation potential, and the capacity to produce paracrine factors, have been demonstrated to play an important role in rescuing injured tissues [5]. Stem cells have been successfully used in the restoration of ovarian function in a cyclophosphamide (CTX)-induced POI mouse model [6–10]. However, their cellular and molecular mechanisms have not been fully elucidated. Moreover, a growing amount of evidence indicates that stem cell therapy may have certain adverse effects, including a high risk of cancer and autoimmune disease [11–13].

Microvesicles (MVs), small vesicles with a diameter of < 1 μm carrying membrane and cytoplasmic constituents of cellular origin, can transfer proteins, mRNA, and bioactive lipids to target cells through surface-expressed ligands and surface receptors, which affect the phenotype and function of the target cells [14, 15]. It has been reported that MVs released from mesenchymal stem cells (MSCs) exert a protective effect on tissues and stimulate tissue repair in vitro and in vivo, and MV transplantation is a novel cell-free therapeutic strategy for various degenerative disorders [16–18]. In this study, we aimed to investigate (i) the effects of human umbilical cord MSC-derived MVs (HUCMSC-MVs) on the functional recovery of injured ovaries in a POI mouse model and (ii) the underlying mechanisms.

## Materials and methods

### Experimental animal

Specific pathogen-free (SPF) grade CD1 (ICR) female mice between 4 and 5 weeks of age were housed under a 12-h light-dark cycle at 25 °C ± 2 °C with food and water provided ad libitum, and all animal experiment protocols were approved by the Ethics Committee of Anhui Medical University.

### Isolation and identification of HUCMSC-MVs

HUCMSCs were obtained from Cell Therapy Center of 105th Hospital of the People’s Liberation Army (PLA) and cultured in α-MEM (Gibco, USA) containing 10% fetal bovine serum (FBS, Sijiqing, China) at 37 °C and 5% CO_2_. When reaching 70–80% confluence, the culture medium was replaced with α-MEM deprived of FBS, then the cells were cultured for 48 h. MVs were obtained from the supernatant of cultured HUCMSCs according to the protocol [19]. The amount of MVs was determined by measuring the total MV-associated proteins, using the Bradford assay. The MVs dissolved in PBS were loaded on copper grids and stained with 1% (*w*/*v*) phosphotungstic acid (PTA) and then observed under a transmission electron microscope (FEI, USA). The protein expressions of CD9, CD63, TSG101, and CANX in the HUCMSC-MVs obtained in this study were compared with UC-MSCs by western blot.

### Establishment of mice premature ovarian insufficiency model

Four- to 5-week-old female ICR mice (*n* = 8) with a normal estrous cycle were used to establish POI model, and the other 8 were served as normal control. To build the POI model, busulfan (20 mg/kg, Sigma-Aldrich, USA) and CTX (200 mg/kg, Sigma-Aldrich, USA) dissolved in dimethyl sulfoxide (DMSO) (Sigma-Aldrich, USA) were administered through single intraperitoneal injection, while the subjects in normal control were injected with DMSO only instead of CTX/BUS. Vaginal smears were obtained at 9:00 am daily and stained with hematoxylin and eosin (HE) for analysis of the estrous cycle. The POI mouse model was evaluated 2 weeks later.

### Detection of transplanted HUCMSC-MVs in the ovaries

Another 20 mice were enrolled to analyze the localization of the HUCMSC-MVs in the ovary, with 10 for POI model and 10 for normal control, respectively. To detect the HUCMSC-MVs in the ovary, 150 μg PKH26-labeled (Sigma-Aldrich, USA) HUCMSC**-**MVs were injected into the mice of POI model and normal control via the vena caudalis. The ovaries of 2 mice from each group were taken at 12 h, 24 h, 48 h, 72 h, and 1 week; the frozen sections of the ovarian tissue were stained with human follicle-stimulating hormone receptor (FSHR) (Abcam, USA) for cytoplasm, while the nuclei were stained with diamidino-phenyl-indole (DAPI, Beyotime, China). The localization of PKH26-labeled HUCMSC**-**MVs in ovarian tissues was observed by confocal laser scanning microscopy.

### Intervention of POI model with HUCMSC-MVs

After the POI model was successfully built, other 24 experimental mice were recruited for the evaluation of the therapeutic efficacy of HUCMSC-MVs on POI mice. They were assigned into normal control group, POI group, and POI-MVs group, with 8 mice for each group, and received different interventions. Briefly, the mice were put in the special container (50 ml test tube with several free breath holes), and their tails leaked out. The tail was disinfected with 75% alcohol, then 100 μl of PBS containing 150 μg MVs was injected into the vena caudalis of the mice with 1 ml syringe for the POI-MVs group, while an equivalent of PBS was sham injected into the vena caudalis of the mice in the control group and POI group. The operation was repeated once a week. Four weeks later, assessment of ovarian function and histomorphometric analysis were carried out for each group (Fig. 5a).

### Histomorphometric analysis of the ovarian follicles and vessels

The ovaries from the normal control group, POI group, and POI-MVs group were fixed with 4% paraformaldehyde (4 °C, overnight), dehydrated through a grade ethanol series, vitrified in xylene, embedded in paraffin, serially sectioned at 5 μm, and then stained with hematoxylin and eosin (HE). The sections were examined and photographed with the Olympus BX-51 light microscope (Olympus, Japan) [20]. The number of follicles including non-atretic, atretic primordial, developing, and preovulatory follicles in each ovary was counted in every randomly selected tenth section. Only the follicles containing a round oocyte with a clearly visible nucleus were scored as healthy, while the ones convoluted, condensed, or fragmented were scored as atretic [21, 22]. Blood vessel counting was carried out at 200-fold field, and five random fields from three different sections were chosen for each sample.

### Immunohistochemical analysis

For the observation of angiogenesis in the ovary, the ovarian section was immunostained with CD34 antibody, a marker for newly formed vascular endothelial cells. Briefly, the ovarian tissue section was incubated with rabbit anti-mice CD34 antibody (Abcam, USA, diluted1:150) overnight at 4 °C, followed by goat anti-rabbit IgG secondary antibody (Abcam, USA, 1:200) for 60 min, then stained with DAB Substrate Kit (Beyotime, China). Hematoxylin was used to co-stain the nuclei. The number of CD34-positive cells was determined by counting five random fields of three different sections from each sample.

### Hormone assay

Blood sample was collected via the orbital artery of mice after anesthetized with inhaled isofurane (#B506, Abbott, Chicago, IL) and centrifuged at 1600 rpm for 10 min. The serum was stored at − 20 °C until measurement. The levels of estradiol (E2) and follicle-stimulating hormone (FSH) were measured with ELISA Kit (Cusabio, China).

### Real-time PCR and western blot

Total RNA of mice ovarian tissue was isolated with Trizol Reagent (Invitrogen, USA). cDNA was synthesized using PrimeScript®RT Master Mix (Takara Bio, Japan) according to the manufacturer’s instructions. qPCR was performed in ABI 7500 real-time PCR system (Applied Biosystems, USA) using SYBR®Premix Ex Taq™ II Kit (Takara Bio, Japan). Primers of vascular endothelial growth factor (*VEGF*), type 1 insulin-like growth factor (*IGF-1*), and *Angiogenin* were designed to span the introns by Roche Universal ProbeLibrary (Table 1), and the specificity and efficiency were confirmed by melting curve and agarose electrophoresis before RT-qPCR. Relative quantification of the gene expression was calculated using the 2-ΔΔCt method normalized to β-actin.

**Table 1:** Primers used for qPCR validation

Gene	Primer FW	Primer RW
*VEGF*	CAGGCTGCTGTAACGATGAA	GCTTTGGTGAGGTTTGATCC
*IGF-1*	GACCGAGGGGCTTTTACTTC	CATCCACAATGCCTGTCTGA
*Angiogenin*	CCAGGCCCGTTGTTCTTGAT	GGAAGGGAGACTTGCTCATTC

Protein of mice ovarian tissue was separated in 12% SDS-PAGE gels and transferred to PVDF membranes blocked with 5% non-fat milk solution then probed with antibodies against VEGF (1:2000, ThermoFisher, USA), total AKT (1:2000, CST, USA), p-Akt (1:2000, CST, USA), and β-actin (1:3000, Kangcheng, China). The bands were visualized on X-ray films using ECL reagents (Thermo Fisher, USA).

### Statistical analysis

The data are presented as the means ± SEM. The parametric variables were compared by Student’s *t* test and analysis of variance. The non-parametric variables were compared by the Mann-Whitney *U* test. Categorical variables were compared by the *χ*^2^ test. *P* < 0.05 was considered statistically significant.

## Results

### Characterization of HUCMSC-MVs

**Fig. 1:**
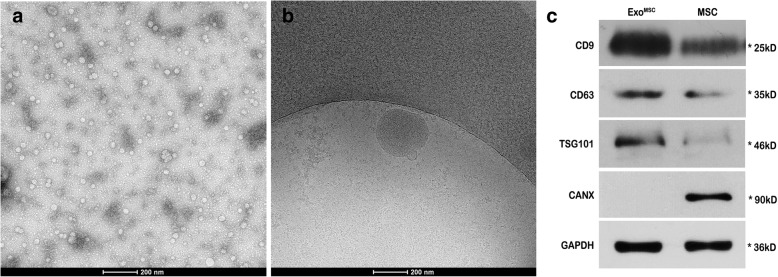
Characterization of MVs. **a**–**b** Morphological features of MVs under an electron microscope. Scale bar = 200 nm. **c** The protein expressions of CD9, CD63, TSG101, and CANX in the HUCMSC-MVs obtained in this study were compared with UC-MSCs by western blot. *EXO*^*MSC*^ HUCMSC-MVs, *MSC* mesenchymal stem cell

By electron microscopy, HUCMSC-MVs were characterized as round-shaped membrane vesicles of approximately 30–200 nm in diameter (Fig. 1a, b). The total protein content of MVs released in 48 h by 10^6^ HUCMSCs under serum-free condition was 162.66 ± 17.50 μg. Western blot showed that the HUCMSC-MVs obtained in this study were highly positive for CD9, CD63, and TSG101 and negative for CANX (Fig. 1c), suggesting they originated from MSCs.

### Evaluation of the established POI model

**Fig. 2:**
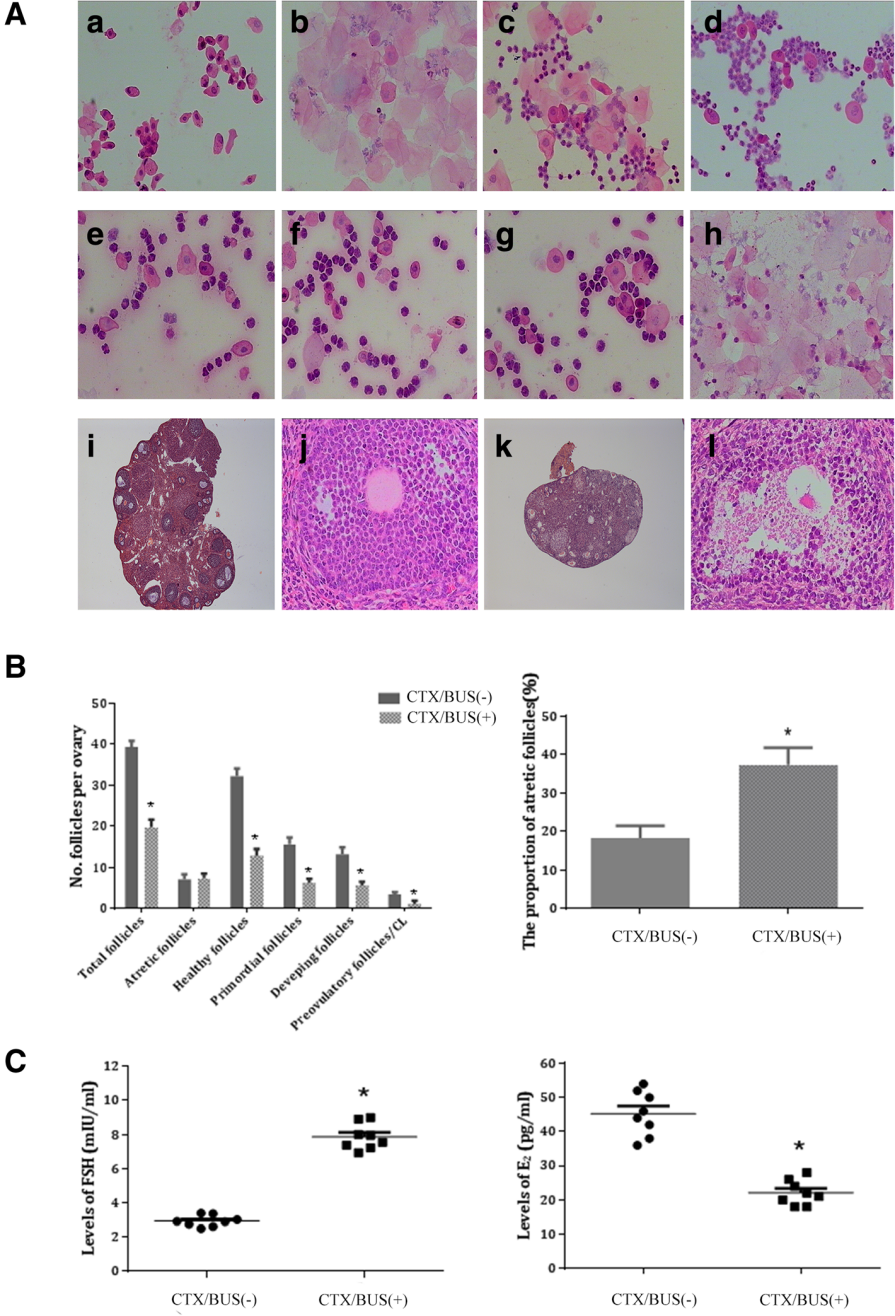
Chemotherapy-induced premature ovarian insufficiency (POI) mice model. **A** Vaginal exfoliative cell smear and ovary section of normal mice (a–d, × 100; i, × 40; j, × 200) and POI mice (e–h, × 100; k, × 40, l, × 200). **B** Comparison of the follicles and the proportion of atretic follicles between CTX/BUS+ group and CTX/BUS− groups (*P < 0.05). **C** E2 and FSH levels determined by ELISA 14 days after CTX/BUS administration (*P < 0.05). *E2* oestradiol, *FSH* follicle-stimulating hormone, *CTX/BUS+* CTX/BUS administration, *CTX/BUS−* CTX/BUS non-administration

The body weight of POI mice was significantly lower than that in the control group. POI mice had an irregular estrous cycle; they exhibited a shorter proestrus and estrus and a longer diestrus [Fig. 2A (e–h)]. The total number of follicles, including atretic, healthy, primordial, developing, and preovulatory follicles, was significantly lower (*P* < 0.05), while the proportion of the atretic follicles in the POI group was significantly higher than that in the control group (*P* < 0.05; Fig. 2B). In addition, degeneration of oocytes, severe interstitial fibrosis in ovarian tissue, hypoestrogenism, and increased levels of FSH were observed in POI mice [Fig. 2A (k, l) and C]. Collectively, these results suggest ovarian dysfunction in POI mice.

### Location of HUCMSC-MVs in the ovary

**Fig. 3:**
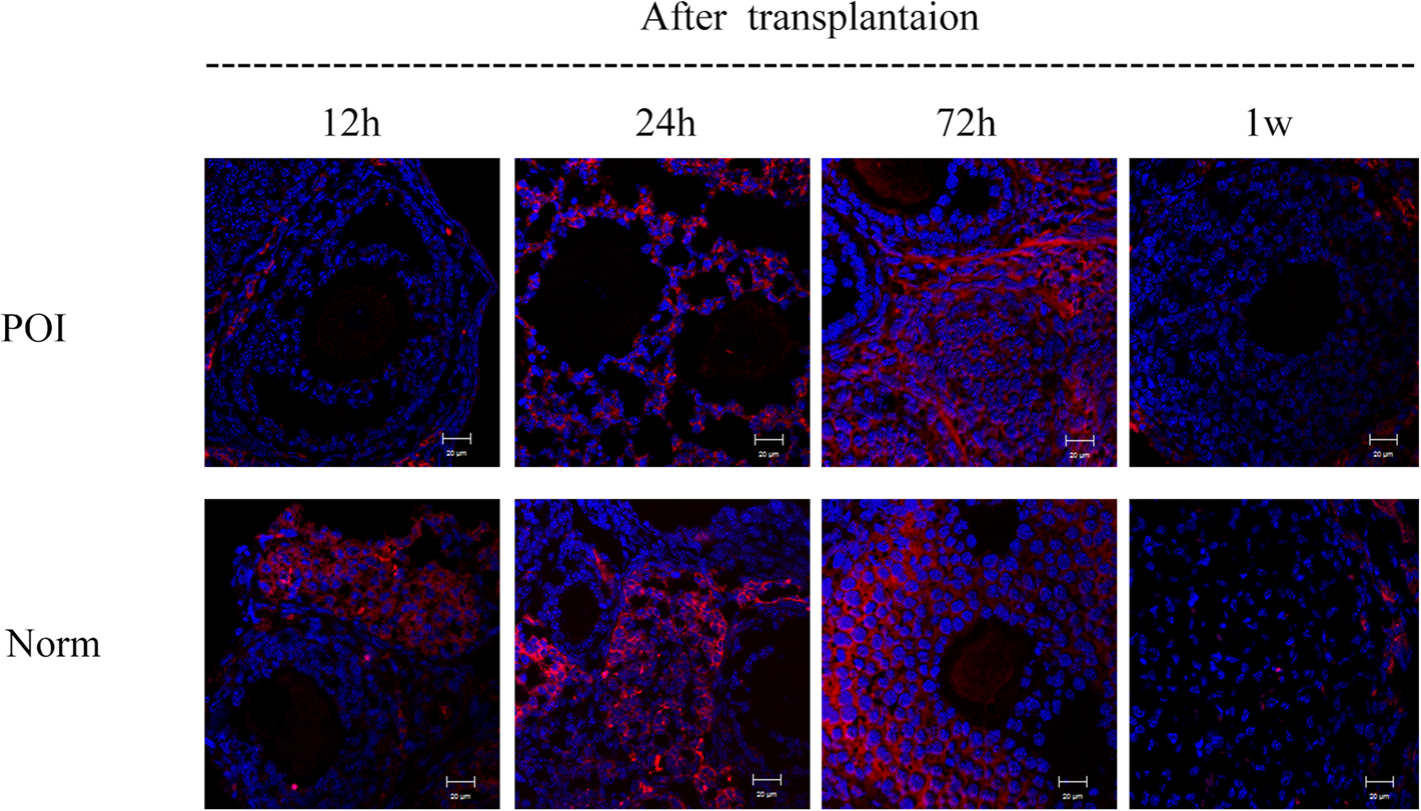
Localization of HUCMSC-MVs in mouse ovaries. PKH26-labeled MVs localization in the ovary tissue of POI mice and control mice. The nuclei were stained with DAPI (blue). Red for PKH26-labeled MVs. Scale bars = 20 μm*. POI* the ovary of POI mice, *Norm* the ovary of normal mice

PKH26-labeled MVs were detectable in ovarian tissue of both POI mice and normal mice 12 h after administration; the fluorescence intensity in the ovarian tissue was markedly enhanced, and PKH26 was taken up into the cytoplasm of granulosa cells (GCs) 24 h later. The signals remained strong for at least 72 h and disappeared 7 days later (Fig. 3).

### HUCMSC-MVs transplantation improved body weight and estrous cycle of POI mice

**Fig. 4:**
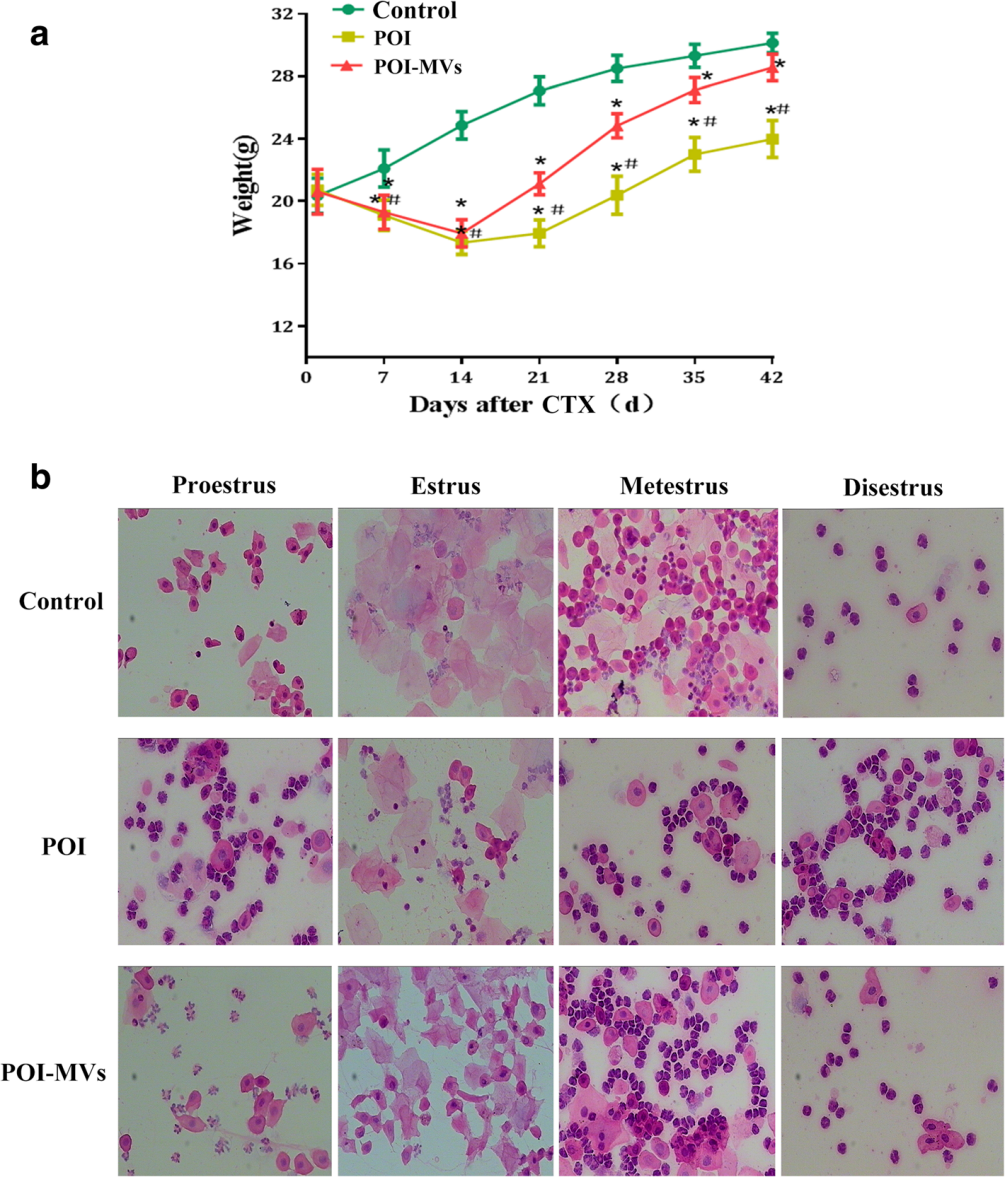
HUCMSC-MVs transplantation improved the body weight and estrous cycle of POI mice. **a** Comparison of the body weight among the three groups (*P < 0.05 vs the control group, ^#^*P* < 0.05 vs the POI-MVs group)*.* **b** Comparison of the estrous cycle among the three groups (× 100)

The mice were weighed once a week after CTX/BUS administration. The body weight of POI-MVs mice was increased significantly after 2 weeks (*P* < 0.05), although it was still lower than that in the control group after 6 weeks (*P* < 0.05; Fig. 4a). The estrous cycle of POI-MVs mice recovered gradually 2 weeks after HUCMSC-MVs transplantation, and 75% of POI-MVs mice showed a regular estrous cycle, including a normal diestrus, proestrus, estrus, and metestrus, 4 and 6 weeks after HUCMSC-MVs transplantation. The estrous cycle of POI mice remained irregular throughout the entire experiment (Fig. 4b).

### HUCMSC-MVs transplantation increased the ovarian follicles of POI mice

**Fig. 5:**
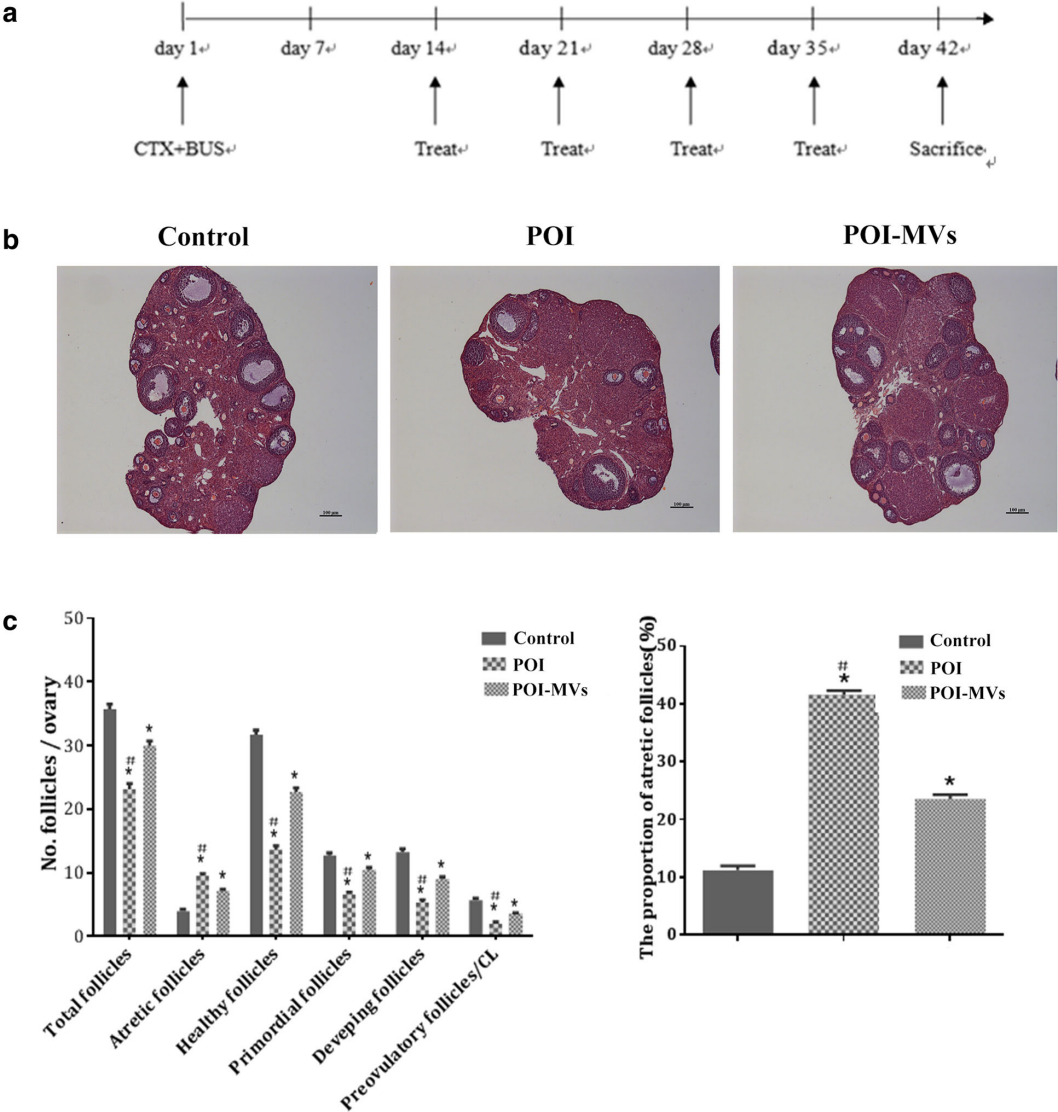
HUCMSC-MVs transplantation in POI mice. **a** The schedule of CTX/BUS administration and treatment with PBS or HUCMSC-MVs. **b** HE staining of the ovarian section 28 days after MVs transplantation. Scale bars = 100 μm. **c** The follicle number and proportion of atretic follicles in the three groups (*P < 0.05 vs the control group, ^#^*P* < 0.05 vs the POI-MVs group)

The numbers of primordial, developing, and preovulatory follicles in POI-MVs mice were higher than those in the POI group (*P* < 0.05). Furthermore, the proportion of atretic follicles was lower, and more healthy follicles were observed in the POI-MVs group (*P* < 0.05). However, the numbers of total, healthy, primordial, developing, and preovulatory follicles in the POI-MVs group were still lower than those in the control group 4 weeks after MVs transplantation (*P* < 0.05; Fig. 5b, c).

### HUCMSC-MVs transplantation improved hormone secretion in POI mice

**Fig. 6:**
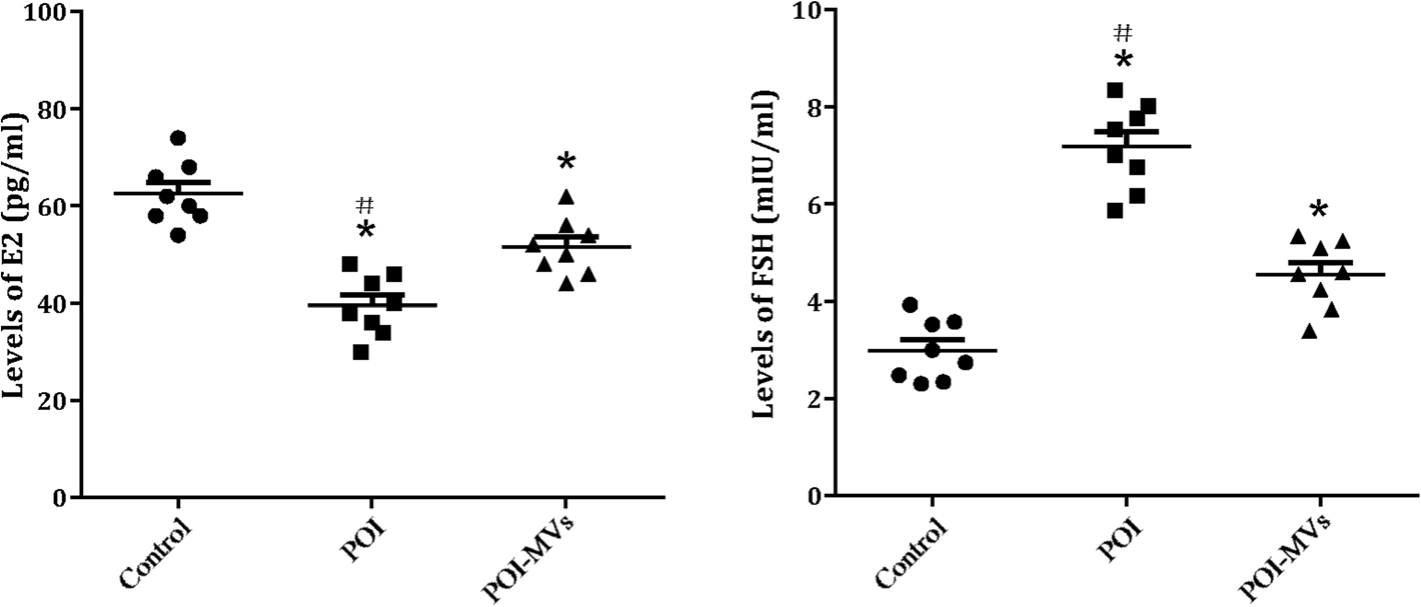
Changes of hormone levels after HUCMSC-MVs transplantation. Serum levels of estradiol (E2) and follicle stimulating hormone (FSH) in three groups (*P < 0.05 vs control group, ^#^*P* < 0.05 vs POI-MVs group

Four weeks after MVs transplantation, the serum level of E2 was significantly higher and that of FSH was significantly lower in the POI-MVs group compared with the POI group (*P* < 0.05), but neither of the two reached the levels of the control group (Fig. 6).

### HUCMSC-MVs transplantation induced angiogenesis and cytokine expression in the ovaries of POI mice

**Fig. 7:**
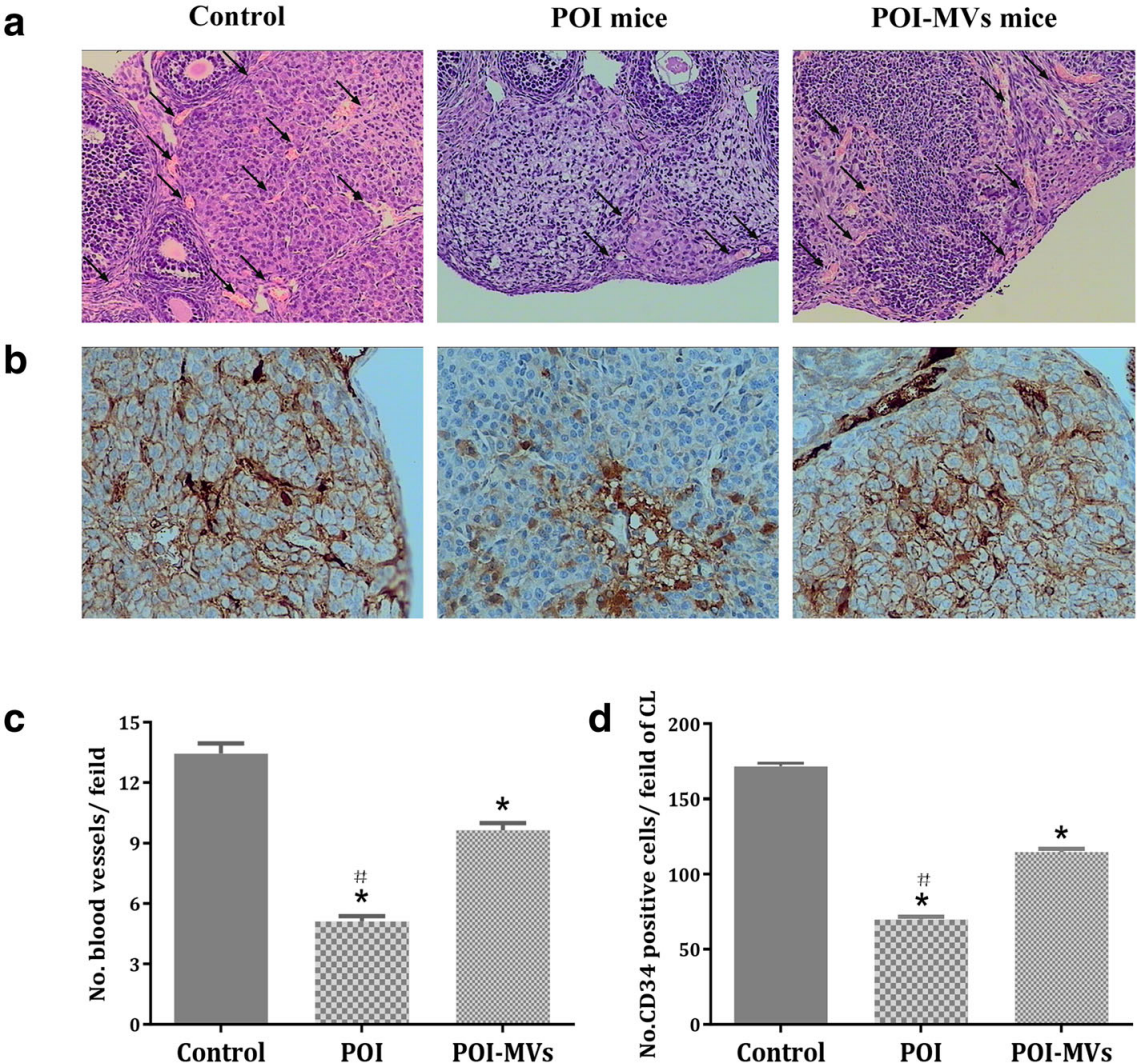
HUCMSC-MVs transplantation promotes ovarian angiogenesis in POI mice. **a** HE staining of the blood vessels (black arrows, × 200). **b** Immunohistochemistry (IHC) of CD34 in the ovaries of the control group, POI group, and POI-MVs group (× 400). **c**, **d** Ovarian blood vessels and CD34-positive cells (*P < 0.05 vs the control group, ^#^*P* < 0.05 vs the POI-MVs group)

**Fig. 8:**
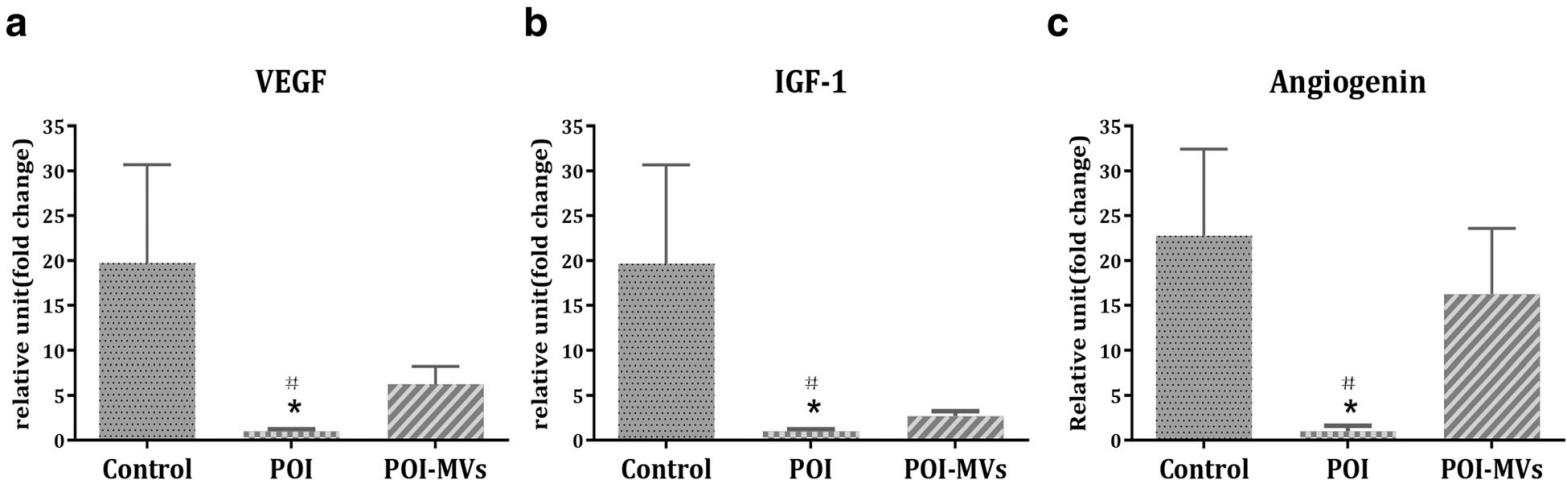
HUCMSC-MVs upregulate the mRNA expression of the cytokine-related angiogenesis in the ovaries of POI mice. The mRNA expression of VEGF (**a**), IGF-1 (**b**), and angiogenin (**c**) in the three groups (*P < 0.05 vs the control group, ^#^*P* < 0.05 vs the POI-MVs group). *VEGF* vascular endothelial growth factor*, IGF-1* type 1 insulin-like growth factor

HE staining showed that there were more cords, tubules, and blood-filled channels containing red blood cells in the ovaries of POI-MVs mice, and the number of blood vessels in the ovaries of POI-MVs mice had increased significantly 4 weeks after HUCMSC-MVs transplantation (*P* < 0.05; Fig. 7a, c). By IHC analysis, we detected that the number of CD34-positive cells in the ovaries of POI mice was significantly lower compared with the control mice (*P* < 0.05). Conversely, more CD34-positive cells were observed in the POI-MVs group (*P* < 0.05; Fig. 7b, d). The RT-PCR results also showed that the mRNA expression levels of angiogenesis-related cytokines, including VEGF, IGF-1, and angiogenin, in the ovaries of POI mice were downregulated significantly, whereas all of those in the POI-MVs group were upregulated significantly compared with the POI group (*P* < 0.05; Fig. 8).

### HUCMSC-MVs transplantation activated the PI3K/AKT signaling pathway in the ovary

**Fig. 9:**
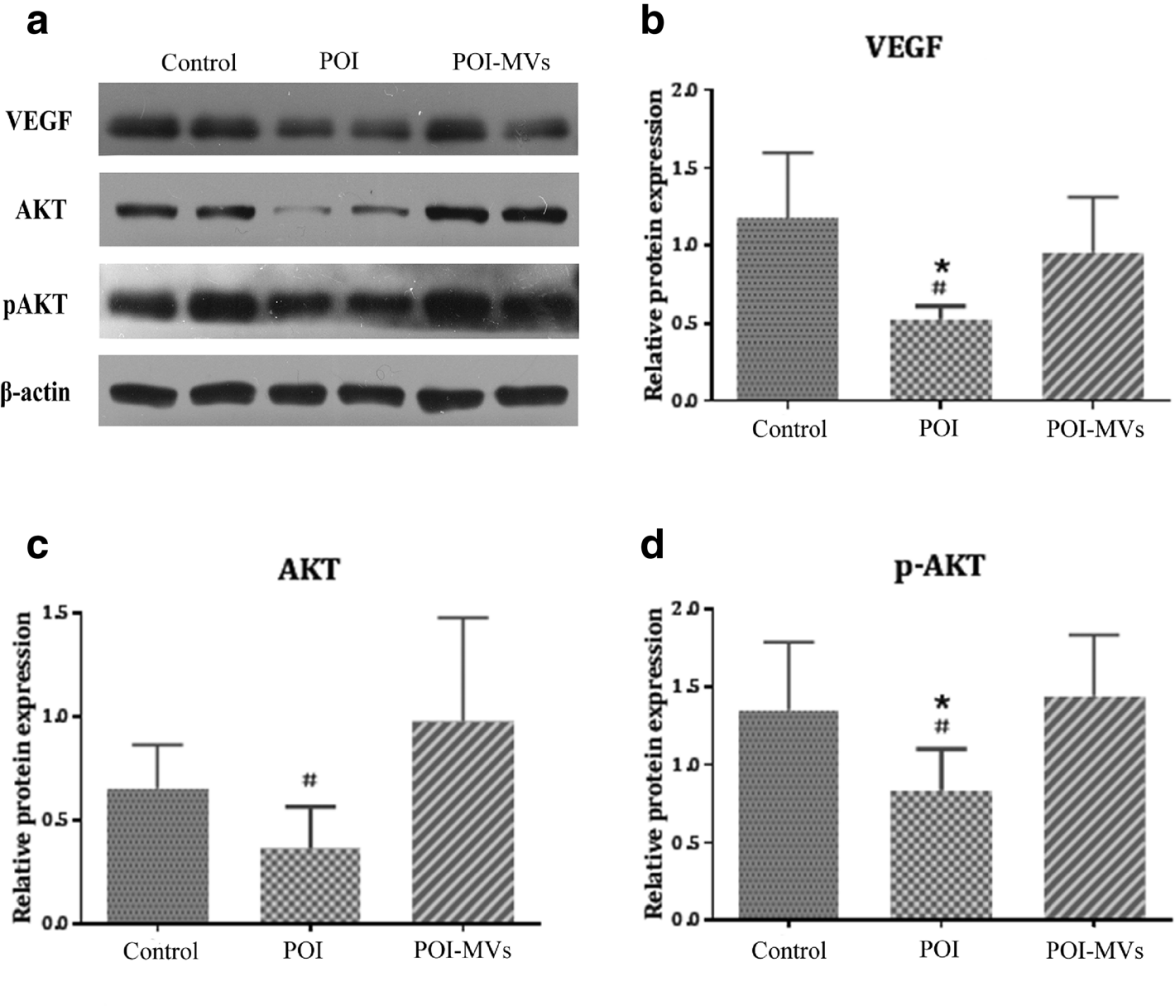
HUCMSC-MVs activate the PI3K/AKT signaling pathway of the ovaries in POI mice. **a** The protein expressions of total-AKT, p-AKT, and VEGF in the ovaries of the three groups. **b** The protein expression of VEGF in the three groups. **c** The protein expression of total-AKT in the three groups. **d** The protein expression of p-AKT in the three groups (*P < 0.05 vs the control group, ^#^*P* < 0.05 vs the POI-MVs group)

The phosphatidylinositol-3-kinase (PI3K)/AKT signaling pathway participates in various cellular biological processes, including angiogenesis, and phosphorylation of AKT is known to be an index of PI3K/AKT signaling pathway activation. The western blot results demonstrated that the expression of total AKT, p-AKT, and VEGF in the ovaries of the POI-MVs group was significantly higher compared with the POI group (*P* < 0.05; Fig. 9).

## Discussion

At present, there is no effective therapy or medication for the treatment of POI. Hormone replacement therapy (HRT), one of the most commonly used treatments for POI, only aims to ameliorate the symptoms caused by the disturbed hormone profile and fails to solve the fundamental problem and restore ovarian function and fertility. Moreover, HRT notably increases the risk of thrombosis and cancer [23, 24]. In recent years, therapeutic effects of stem cells derived from a number of sources, including bone marrow, amniotic fluid, and adipose tissue, on long-term infertility and ovarian damage have been reported [25–27]. However, the disadvantages resulting from stem cell therapy, including ambiguous curative effects, high risk of cancer, and autoimmune diseases, cannot be ignored [11–13].

MVs and extracellular membrane vesicles that can transfer proteins, lipids, and mRNAs from the parent cell to target cells play a key role in intercellular and even interorgan communication [28]. Various cell types can release MVs when they are activated. Among those, MSCs, which exhibit a secretory activity that is at least 10 times higher than that of other cells, are believed to be the main MVs-producing cells [29]. It has been shown that MVs secreted by MSCs can reduce tissue injury through modulating immune responses and promoting self-repair of injured cells [30], and the components of MSC-derived conditioned medium, such as soluble factors, MVs, and potential organelles, possess many therapeutic properties, indicating that MSC engraftment might not be required [31]. Therefore, MVs transplantation, a new cell-free therapy, may provide a novel strategy to treat POI in practice.

GCs are essential components of the ovarian follicles, playing a supportive and regulatory role in the maturation and development of oocytes and aiding in the maintenance of the hormonal balance in the ovarian niche via the autocrine and paracrine mechanisms. To validate that HUCMSC-MVs could target and be internalized into GCs, PKH26-labeled HUCMSC-MVs were injected into the vena caudalis of model mice, and frozen sections of FSHR-positive ovarian tissue (specific for GCs) were observed under a confocal laser scanning microscope. Red fluorescent signals of PKH26-labeled HUCMSC-MVs were detected in GCs 24 h after transplantation, and the signals remained strong for at least 72 h, indicating that HUCMSC-MVs could target and be internalized into both POI and control mouse ovarian tissue. As the signals almost disappeared 7 days later, the administration of MVs via the vena caudalis in the POI-MVs group was repeated once a week for a month.

Our results show that the disturbed estrous cycle, the levels of E2 and FSH, and the body weight of POI mice exhibited a significant recovery after HUCMSC-MVs transplantation, consistent with a previous study [32]. Moreover, the numbers of primordial, developing, and preovulatory follicles in the ovaries of the POI-MVs group were significantly higher, and the numbers of healthy and atretic follicles were similar to those in the control group 4 weeks after HUCMSC-MVs transplantation. Collectively, these results indicate that HUCMSC-MVs transplantation could prevent follicle atresia and restore the ovarian function of CTX/BUS-induced POI mice.

Ovarian function and follicle development strongly depend on the establishment and continuous remodeling of a complex vascular system in the ovary, which enables the follicle and/or corpus luteum to receive the required supply of nutrients, oxygen, and hormonal support and to synthesize and release steroids [33, 34, 35]. Besides, the formation of a rich vascular network in the primary follicles is considered to be responsible for the selection of the dominant follicle. It has been reported that transplantation of human adipose-derived MSCs could restore damaged ovarian function through inducing angiogenesis and improving the number of ovarian follicles and corpora lutea [8]. In this study, we first illustrated that the number of blood vessels and CD34-positive cells in the ovaries of POI-MVs mice was significantly higher 4 weeks after HUCMSC-MVs transplantation, indicating that MVs transplantation could induce angiogenesis and promote the formation of new blood vessels in the chemotherapy-injured ovary.

Angiogenic factors play important roles in various aspects of neovascularization, including cell proliferation, differentiation, migration, and morphogenesis of vascular cells, and MVs derived from HUCMSCs contain many angiogenesis promoting biomolecules, such as angiogenin, VEGF, VEGF-R2, and MCP-1; some of these are present at higher levels in MVs than in parent cells [36]. VEGF, IGF-1, and angiogenin are well-known angiogenic factors participating in the regulation of follicular development through vascular-independent mechanisms [37–40]. Our results show the mRNA expression levels of VEGF, IGF-1, and angiogenin in the ovaries of POI-MVs mice are significantly higher than those in POI mice, suggesting that high expression of angiogenic factors induced by HUCMSC-MVs might play a critical role in the restoration of ovarian function.

As is well known, the PI3K-AKT pathway, a key signaling pathway implicated in angiogenesis, regulates the expression of downstream angiogenic factors such as VEGF [41, 42]. Hung et al. found that MSC-conditioned medium under hypoxic conditions could inhibit apoptosis of endothelial cells and stimulate angiogenesis via activation of the PI3K-AKT pathway [43]. Moreover, Jia et al. confirmed that bone marrow mesenchymal stromal cell transplantation could ameliorate angiogenesis and reduce renal damage in rats by activating the PI3K-AKT signaling pathway and increasing serum VEGF level [41]. In this study, we demonstrated that the expression levels of total AKT, p-AKT, and VEGF in the ovary of POI-MVs mice were significantly higher than those in POI mice, indicating that HUCMSC-MVs transplantation might activate the PI3K-AKT signaling pathway and promote VEGF expression. The mechanism by which MVs activate the AKT signaling pathway will be the subject of future research.

There are some limitations in this study. Firstly, a natural mating trial would provide supplementary evidence to prove the effect of MVs in restoring damaged ovarian function; these experiments have been carried out and are described in another publication. Secondly, the biggest challenge for MVs therapy may be the exploration of molecular mechanisms involved in the angiogenesis-promoting effects, which will be the subject of our future work.

Considering the human clinical application of MVs, several concerns remain to be addressed. Firstly, there are two ways of transplanting MVs, that is, the local injection of MVs into the ovarian tissue or into the blood circulation. We have not compared the efficiency of these two methods in our study. Secondly, as is well known, angiogenesis plays a critical role in tumor growth, and it is uncertain whether high doses of MVs can cause serious adverse effects, such as ovarian cancer. Therefore, more work on the efficiency and safety of the in vivo use of MVs must be done before it can be applied in clinical practice.

## Conclusions

We first demonstrated that HUCMSC-derived MVs transplantation could restore ovarian function of POI mice model via activation of the PI3K-AKT signaling pathway and promoting angiogenesis of the damaged ovary, which may provide a potential therapeusis for the patients with POI.

## Data Availability

The datasets used and/or analyzed during the current study are available from the corresponding author on reasonable request.

## Abbreviations

AKT: Serine/threonine kinase
BUS: Busulfan
CTX: Cyclophosphamide
DAPI: Diamidino-phenyl-indole
DMSO: Dimethylsulfoxide
E2: Estradiol
FSH: Follicle-stimulating hormone
GCs: Granulosa cells
HRT: Hormone replacement therapy
HUCMSC-MVs: Human umbilical cord mesenchymal stem cell-derived microvesicles
IGF-1: Type 1 insulin-like growth factor
MSC: Mesenchymal stem cells
MVs: Microvesicles
PI3K: Phosphatidylinositol 3-kinase
POI: Premature ovarian insufficiency
VEGF: Vascular endothelial growth factor
